# An independent assessment of an artificial intelligence system for prostate cancer detection shows strong diagnostic accuracy

**DOI:** 10.1038/s41379-021-00794-x

**Published:** 2021-03-29

**Authors:** Sudhir Perincheri, Angelique Wolf Levi, Romulo Celli, Peter Gershkovich, David Rimm, Jon Stanley Morrow, Brandon Rothrock, Patricia Raciti, David Klimstra, John Sinard

**Affiliations:** 1 Department of Pathology, Yale School of Medicine, New Haven, CT USA; 2 Paige.AI, 11 Times Square, New York, NY USA; 3 Department of Pathology, Memorial Sloan Kettering Cancer Center, New York, NY USA; 4 Present Address: Department of Pathology, Middlesex Hospital, Middletown, CT USA

**Keywords:** Pathology, Prostate cancer

## Abstract

Prostate cancer is a leading cause of morbidity and mortality for adult males in the US. The diagnosis of prostate carcinoma is usually made on prostate core needle biopsies obtained through a transrectal approach. These biopsies may account for a significant portion of the pathologists’ workload, yet variability in the experience and expertise, as well as fatigue of the pathologist may adversely affect the reliability of cancer detection. Machine-learning algorithms are increasingly being developed as tools to aid and improve diagnostic accuracy in anatomic pathology. The Paige Prostate AI-based digital diagnostic is one such tool trained on the digital slide archive of New York’s Memorial Sloan Kettering Cancer Center (MSKCC) that categorizes a prostate biopsy whole-slide image as either “Suspicious” or “Not Suspicious” for prostatic adenocarcinoma. To evaluate the performance of this program on prostate biopsies secured, processed, and independently diagnosed at an unrelated institution, we used Paige Prostate to review 1876 prostate core biopsy whole-slide images (WSIs) from our practice at Yale Medicine. Paige Prostate categorizations were compared to the pathology diagnosis originally rendered on the glass slides for each core biopsy. Discrepancies between the rendered diagnosis and categorization by Paige Prostate were each manually reviewed by pathologists with specialized genitourinary pathology expertise. Paige Prostate showed a sensitivity of 97.7% and positive predictive value of 97.9%, and a specificity of 99.3% and negative predictive value of 99.2% in identifying core biopsies with cancer in a data set derived from an independent institution. Areas for improvement were identified in Paige Prostate’s handling of poor quality scans. Overall, these results demonstrate the feasibility of porting a machine-learning algorithm to an institution remote from its training set, and highlight the potential of such algorithms as a powerful workflow tool for the evaluation of prostate core biopsies in surgical pathology practices.

## Introduction

Prostate cancer is a leading cause of cancer-related morbidity and mortality in the US. It is estimated that 191,930 new cases of prostate cancer will be diagnosed in the US in 2020 accounting for 10.6% of all new cancer cases. Prostate cancer is also predicted to cause 33,330 deaths in 2020 accounting for 5.5% of all cancer deaths [1]. The diagnosis of prostate cancer is made on tissue biopsies typically triggered by elevations of prostate-specific antigen and/or an abnormal digital rectal exam. Traditionally prostate core biopsies have been obtained by systematic biopsy of the peripheral zone of the prostate. In recent years, multiparametric magnetic resonance imaging of the pelvis has increasingly been used to detect prostatic lesions and to obtain targeted core biopsies of the lesions in addition to the traditional systematic cores [2]. As a result, prostate core biopsies are often a significant portion of the anatomic pathologists’ work load in many surgical pathology settings. The accurate detection, quantitation and grading of prostate cancer is critically important in the evolving landscape of prostate cancer treatment [3–6]. While blinded re-review of slides can increase cancer detection and accuracy, time and workflow considerations impede routine adoption of second-reads in surgical pathology practices [7–9]. Computer-aided diagnosis with the potential to improve the accuracy of prostate core biopsy diagnoses while simultaneously decreasing turnaround times and relieving pathologist workload would be enormously useful in surgical pathology practice.

The evolution and adoption of digital pathology technologies is predicted to improve diagnostic accuracy in the anatomic pathology realm [10]. Machine-learning algorithms applied on digitized images have demonstrated accurate performance in identifying disease features and phenotypes [11, 12]. Ideally, in order to have diagnostic utility, machine-learning algorithms in anatomic pathology should demonstrate comparable performance across whole-slide imaging (WSI) datasets regardless of the origin of the images. Paige Prostate is a machine-learning algorithm trained on the digital slide archive of Memorial Sloan Kettering Cancer Center (MSKCC) in New York that takes a whole-slide image as input and categorizes the image as either “suspicious” for prostatic adenocarcinoma if the algorithm detects adenocarcinoma or glandular atypia (including focal glandular atypia (FGA), high-grade prostatic intraepithelial neoplasia with adjacent atypical glands (PIN-ATYP) or atypical small acinar proliferation (ASAP)); or “not suspicious” for prostatic adenocarcinoma if none of these lesions are detected [13]. In this study, we evaluated the performance of Paige Prostate on a prostate core biopsy WSI data set from Yale Medicine that the algorithm had not previously seen. The study was designed to investigate two potential use case scenarios for Paige Prostate: (1) its utility as a prescreening tool to identify negative cores not requiring manual review by a pathologist and (2) its utility as a second read tool to identify cancer foci not identified by the pathologist. Ideally, in the former use case, all cores categorized as “not suspicious” by the algorithm would be free of tumor, decreasing the number of cores needing pathologist review and, therefore, reducing turnaround time and increasing pathologist productivity. In the latter use case, the algorithm would be used to detect missed foci of cancer, increasing accuracy of reads and thus impacting assessment of tumor volume and location within the prostate, which are important metrics used in deciding between active surveillance versus curative treatments, or for targeting precision radiation therapy for clinical management.

## Materials and methods

### Sample selection and scanning

A total of 1876 prostate core biopsies from 118 consecutive patients procured at Yale Medicine and processed at Yale Pathology from June to July 2019 were included in the analysis. Because of the institutional use of MRI/Ultrasound fusion for guidance of targeted prostate biopsies at Yale, there were often in excess of 20 separately identified prostate core biopsies from each patient. Per institutional policy, for routine diagnostic purposes five histologic levels were prepared from each core biopsy. Levels 1, 3 and 5 were stained with hematoxylin and eosin and levels 2 and 4 were left unstained for possible immunohistochemical staining. The clinically reported discrete diagnosis for each core biopsy rendered by a board certified pathologist after review of all H&E stained levels with additional immunohistochemical (IHC) workup as needed was treated as the ground truth diagnosis. Level 3 of each core biopsy was scanned using a Leica AT-2 WSI scanner® (Leica Biosystems, IL) at a 20× magnification. Scans which the scanner software identified as having failed were repeated; no other quality assurance step was performed on the scans. Scanned images were stripped of identifiers and provided to Paige for processing with the Paige Prostate algorithm. The algorithm was applied without any site-specific adjustments or tuning. The version of software used in this study differs from the originally described version [13] in efficiency and design of the underlying software; the categorization algorithm produces identical results to the original version.

### Analysis

**Fig. 1:**
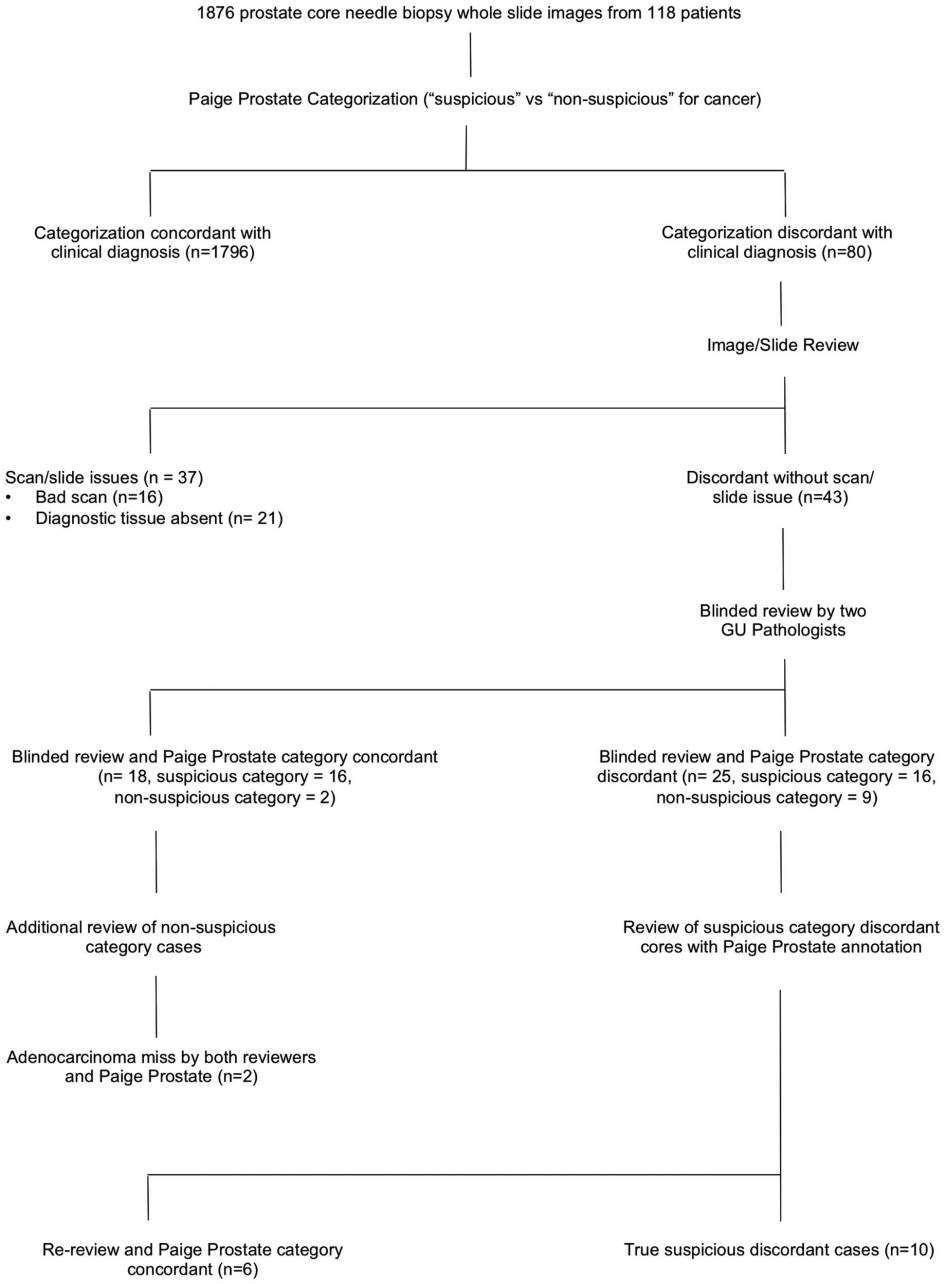
Study design. Flow diagram summarizing the analytic pipeline of the study.

The algorithm categorized each core biopsy as “suspicious” if the algorithm detected adenocarcinoma or glandular atypia (including FGA, PIN-ATYP and ASAP) and as “not suspicious” if none of those lesions were detected. Of note, the algorithm treats high-grade prostatic intraepithelial neoplasia (HG-PIN) as not suspicious. In addition, Paige Prostate flags slides as being out of distribution (OOD) if the thumbnail image (224 × 224 pixels) is significantly different from the distribution of core needle biopsy slides used to develop the algorithm as a way of indicating to pathologists that the incoming data are significantly different than it expects. Common causes of OOD flags include no prostate tissue present, cracks or bubbles, and marker strokes. Core biopsies for which the algorithm’s categorization differed from the rendered diagnosis were treated as tentatively discrepant. Digital images and/or glass slides of discrepant biopsies were reviewed to confirm the presence of diagnostic lesional tissue on the scanned level. Tentatively discrepant biopsy images were intermixed with thirty randomly selected core biopsy images and the whole-slide images were manually reviewed independently by two genitourinary pathologists blinded to the algorithm categorization and the previously rendered final diagnosis. The thirty intermixed cases included 22 core biopsies with a final diagnosis of benign prostatic tissue, 6 cores with prostatic adenocarcinoma, one with FGA and one with HG-PIN. Any discrepancy in manual reads was resolved by consensus except for cores where either of the two pathologists diagnosed glandular atypia. Core biopsies categorized as “not suspicious” by the algorithm but with a final rendered diagnosis of glandular atypia were classified as non-discrepant if both reviewers rendered a benign diagnosis and discrepant if either of the two reviewers diagnosed atypia. Core biopsies categorized as “suspicious” by the algorithm were classified as non-discrepant if either of the two reviewers diagnosed atypia and discrepant if neither of the two reviewers diagnosed atypia. For core biopsies that remained discrepant after blinded review, images were manually rereviewed by the same pathologists with the algorithm highlighting the focus of interest. The analytic pipeline is summarized as a flow diagram in Fig. 1.

## Results

Of the 118 patients represented by the 1876 core biopsies in the study, 86 patients were diagnosed with prostatic adenocarcinoma in at least one core (range of 1–15 positive cores per patient) while 32 patients were diagnosed not having cancer (see Table 1 for brief clinical characteristics). Paige Prostate’s analysis categorized at least one core as suspicious in 84 of the 86 patients with adenocarcinoma while no cores were categorized as suspicious in 26 of the 32 patients without carcinoma or glandular atypia. Of the 1876 core biopsies, 489 cores had a discrete diagnosis of adenocarcinoma, PIN-ATYP, ASAP or FGA; the rest of the cores were diagnosed as benign, high-grade PIN, or as having no prostatic glandular tissue present (see Table 2). There was an apparent discrepancy between the final diagnosis and Paige Prostate categorization in 80 cores. Of these, 46 cores were further analyzed as “not suspicious discrepant cores” (Paige categorization “not suspicious” but final diagnosis was adenocarcinoma, PIN-ATYP, ASAP or FGA) and 34 were further analyzed “suspicious discrepant cores” (Paige categorization “suspicious” but final diagnoses was benign, HG-PIN or no prostatic glandular tissue present).

**Table 1:** Brief summary of clinical characteristics of patient cohort.

Patient characteristics	
Age range (years)	(*N*)
45–50	2
51–60	28
61–70	45
71–80	38
81–90	5
PSA range	0.5–305.5 ng/ml
Highest Gleason Grade	(*N*)
Gleason Grade 3 + 3 = 6/10 (Grade Group 1)	40
Gleason Grade 3 + 4 = 7/10 (Grade Group 2)	21
Gleason Grade 4 + 3 = 7/10 (Grade Group 3)	7
Gleason Grade 4 + 4 = 8/10 (Grade Group 4)	9
Gleason Grade 4 + 5 = 9/10 (Grade Group 5)	8
Gleason Grade 5 + 4 = 9/10 (Grade Group 5)	1
No cancer	32
Prior documented history of adenocarcinoma (on surveillance or treated)	43

**Table 2:** Summary of the clinically rendered diagnoses of 1 876 core biopsies and their corresponding categorization by Paige Prostate.

Final Diagnosis	Number of cores	Paige Prostate Categorization	
		Suspicious	Not suspicious
Carcinoma	438	411	27
Atypia (FGA /ASAP/PIN-ATYP)	51	32	19
HG-PIN	18	6	12
Benign prostatic tissue	1229	26	1203
No prostatic glandular tissue present	140	2	138

### Not suspicious discrepant cores

The 46 not-suspicious discrepant cores yield an apparent negative predictive value of 96.7%/specificity of 97.6% for Paige Prostate. Further analysis revealed that for 16 of these cores the scanned images were not interpretable manually (Table 3): 14 were out of focus, one had separation of coverslip and in one case the tissue was folded on itself. All sixteen cores had a final rendered diagnosis of adenocarcinoma. Seven of the 16 scanned images were flagged as being OOD by the algorithm. In 19 other discrepant cores, diagnostic lesional tissue was not present in the scanned image. Removing the 16 bad scans from the analysis and treating the 19 cores without diagnostic tissue in the scanned image as being concordant leaves 11 truly discrepant cores (5 cores with adenocarcinoma and 6 cores with glandular atypia) yielding a revised negative predictive value of 99.2%.

**Table 3:** Results of manual review of “not suspicious” discrepant cores (*n* = 46). Columns on the right denote the clinically rendered final diagnosis for each core.

Manual review category	Final diagnosis group		Total
	Atypical	Adenocarcinoma	
Agree (Diagnostic tissue not present on scanned level)	13	6	19
Scan Failure	0	16	16
Miss-ASAP	6	0	6
Miss-carcinoma	0	5	5

Since the discrepant carcinoma biopsies constitute the most clinically significant misses, these were investigated in greater detail and included the following:

1. Core biopsy with a 1 mm focus of foamy gland carcinoma confirmed by IHC (Fig. 2A, B). Both blinded reviewers identified this focus. This core was the only core from a 12-core case with carcinoma. Two other cores of this case were diagnosed with HG-PIN and PIN-ATYP; the latter of which was categorized as suspicious by Paige Prostate.

**Fig. 2:**
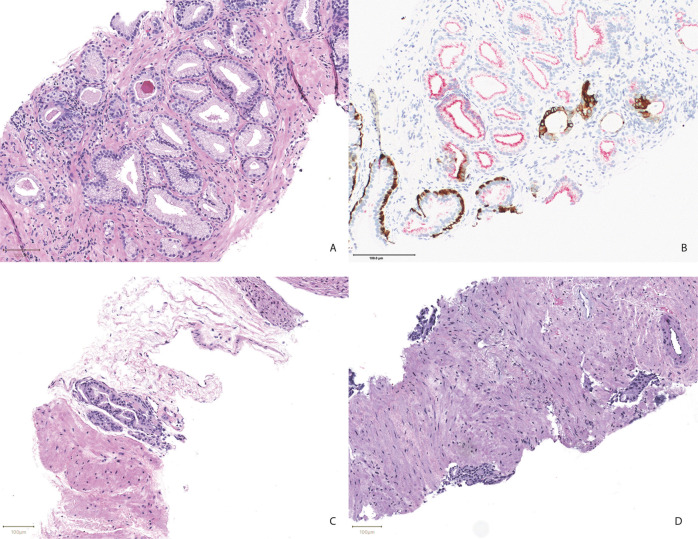
Micrographs of adenocarcinoma foci missed by Paige Prostate. **A** Shows a focus of adenocarcinoma with foamy gland features with the corresponding PIN-4 immunostain in **B**. A focus of perineural adenocarcinoma is shown in **C**. **D** Shows a focus of adenocarcinoma with androgen deprivation therapy effect. (Scale bars = 100 μm).
2. Core biopsy with a 0.5 mm focus of carcinoma showing perineural invasion (Fig. 2C). Both manual reviewers identified this focus. Eight of the 21 remaining cores of this case were diagnosed with carcinoma, all of which were categorized as suspicious by Paige Prostate.
3. Core biopsy with 1 mm focus of carcinoma that was also missed by both manual reviewers and better seen on deeper levels. Nine of the 16 remaining cores of this case were diagnosed with carcinoma, all of which were categorized as suspicious by Paige Prostate.
4. Core biopsy with 0.5 mm of foamy gland carcinoma confirmed by IHC that was also missed by both manual reviewers. Four of the 21 remaining cores of this case were diagnosed with carcinoma, all of which were categorized as suspicious by Paige Prostate.
5. Core biopsy with atypical glands diagnosed as adenocarcinoma with hormone-deprivation therapy effect, spanning 2 mm on deeper levels (Fig. 2D). Both manual reviewers identified atypical glands and indicated that they would request levels. Thirteen of the 26 remaining cores of this case were diagnosed with carcinoma, 12 of which were categorized as suspicious by Paige Prostate and one had a scan failure due to a separated coverslip.

On manual review of the images of six core biopsies with glandular atypia, both reviewers diagnosed atypical glands in five cores and one pathologist diagnosed atypical glands in the remaining core. In five of the six cores, one or both reviewers felt that an IHC workup was further needed for accurate classification.

### Suspicious discrepant cores

Thirty four (34) of the 477 core biopsies categorized as “suspicious” by Paige Prostate had a final diagnosis of benign prostatic tissue, HG-PIN, no prostatic glandular tissue identified or granulomatous prostatitis, yielding an apparent positive predictive value of 92.9%/sensitivity of 90.6% (Table 2). In 32 of these images categorized as suspicious, the largest focus identified by the algorithm was 0.6 mm, and the vast majority (23/32) had foci smaller than 0.2 mm. On manual review, at least one of the reviewers diagnosed atypical glands in 16 cases (Table 4). Two cores had no prostatic glandular tissue present and had been flagged as OOD by the algorithm. The remaining cores were all benign with one core showing granulomatous prostatitis. Treating these 16 cores where at least one of the manual reviewers felt that glandular atypia was present as non-discrepant and removing the two OOD scans from the analysis yields a revised PPV of 96.6%. Paige Prostate has a feature that highlights areas of concern in suspicious cases. When the manual reviewers re-reviewed suspicious discrepant cores with Paige Prostate annotation, 6 additional cores were classified as atypical by at least one of the two reviewers. If these cores are re-classified as non-discrepant, the total ‘suspicious discrepant cores’ become 10; thus, the positive predictive value increases to 97.9%.

**Table 4:** Results of manual review of “suspicious” discrepant cores (*n* = 34).

Final diagnosis group	Manual review category		Total
	Agree	Overcall	
Benign	0	15	15
Atypical	16	0	16
No prostatic glandular tissue	0	2	2
Granulomatous prostatitis	0	1	1

### Manual reads of random intermixed cases

To prevent bias, 30 randomly selected core biopsy images were intermixed with discrepant cores for manual reviewers that included 22 benign cores, one with FGA, six with adenocarcinoma and one with HG-PIN. The manual reads were concordant with the final rendered diagnosis in all cores except for one core with adenocarcinoma where both reviewers diagnosed atypia needing IHC, one core with atypia called benign by both reviewers and one core with a benign diagnosis for which one reviewer diagnosed atypia. The core with HG-PIN was out-of-focus and manually uninterpretable.

At the end of our analysis to determine truly discordant cores and after removing bad scans or scans without lesional tissue, we find that 465 out of 475 “suspicious for cancer” and 1371 out of 1382 “not suspicious for cancer” categorizations by Paige Prostate were concordant. Based on these values Paige Prostate showed positive predictive value of 97.9%, a negative predictive value of 99.2%, sensitivity of 97.7% and specificity of 99.3% in our data set. Identifying the truly discrepant cores based on our analysis yields an F_1_ score of 0.98.

## Discussion

Paige Prostate is a machine-learning algorithm designed to categorize a prostate core biopsy whole-slide image as either “suspicious” or “not suspicious” for prostatic adenocarcinoma. This study was designed to evaluate Paige Prostate on a set of independent scanned slides from Yale Medicine, an academic medical center with a high volume of prostate biopsies where most biopsies are reviewed by a resident/fellow and a subspecialized genitourinary (GU) pathologist, and borderline/tough calls are reviewed in routine consensus conference. In this specific practice setting, the goal of this study was to determine the algorithm’s utility as a prescreening tool to identify cases without carcinoma and as a second read tool to detect manually missed foci of carcinoma without any site-specific calibration of the algorithm. The first use case is a potential productivity tool to allow the pathologist to focus only on cases suspicious for malignancy, allowing greater sign out volume per day. For this scenario, a high negative predictive value is desirable. The second use case is a potential patient safety tool by increasing the accuracy of rendered diagnosis. In this use case, a high positive predictive value is desirable.

In this study if Paige Prostate was used as prescreening tool such that only those cores categorized as suspicious or as out of distribution were manually reviewed, a pathologist would have to review only 589 of 1876 core biopsies (31.4%), substantially increasing productivity. In the absence of any additional quality review of scanned images, this would also mean that 14 cores with adenocarcinoma (5 cores with in-focus images and another 9 cores with scan issue not flagged as being OOD) would be missed, as well as six cores with glandular atypia. Four of the five missed foci of adenocarcinoma were 1 mm or less in size, two of which were also missed by manual reviewers. The missed foci of adenocarcinoma in two of the five in-focus cores had foamy gland features, a variant that often has deceptively benign cytologic features. While these data raise the possibility that the algorithm may have some difficulty with this morphologic variant, we were able to find at least one other instance of carcinoma with foamy gland features that was accurately flagged by Paige Prostate (Supplementary Fig. 1). Four of the five missed malignant cores were from patients who had other cores with larger volume or higher grade disease that was correctly categorized by the algorithm and the misses are unlikely to have had significant clinical impact in these instances. The fifth missed core was from a patient with prior documented low-grade, low-volume disease undergoing routine surveillance and the miss likely would not have changed clinical management.

It is also important to recognize that only one of three levels from each prostate core needle biopsy was scanned for this study. In practice, pathologists examine multiple levels to increase the possibility of detecting a focus of carcinoma or glandular atypia. It is possible that some performance metrics of the algorithm, including its detection of carcinomatous or atypical foci, would be enhanced if all individual core levels had been scanned and were subjected to analysis. It is also possible that these additional levels scanned might not suffer from the same scanning artifacts that compromised the level scanned for algorithmic analysis in this study.

The impact of missing 9 cores with adenocarcinoma that had scan issues but were not flagged as being OOD highlights the importance of additional stringent scan quality assurance steps while using machine-learning diagnostic tools in anatomic pathology. In this study, it would have meant that at least two patients with carcinoma would have been rendered a benign diagnosis. The impact of the missed glandular atypia/ASAP cores is more difficult to characterize. This diagnostic group includes cores with atypical glands where the focus of concern does not meet morphologic threshold for an outright diagnosis of carcinoma. The atypical glands may represent an under-sampled tumor or benign mimics of malignancy such as partial atrophy. ASAP is reported in ~3% of prostate core biopsies and is associated with a higher risk of finding adenocarcinoma on re-biopsy [14, 15]. While these lesions are not actionable from the urologist’s standpoint, from the pathologist’s perspective this can prompt review of additional levels and immunohistochemical stains. This is best highlighted by the missed carcinoma “not-suspicious” discrepant core #5 (above) in this study where both manual reviewers diagnosed glandular atypia that was revealed to be a larger focus of adenocarcinoma on levels. Overall, if we consider cases without scan issues, Paige Prostate flagged at least one core of a case as suspicious in patients with carcinoma highlighting its potential as a screening tool, at least on a per case basis.

If the algorithm had been used as a “second read” on slides that were called benign by the pathologist to verify that no carcinoma had been missed, only 34 slides (1.8% of total) would be categorized as suspicious. The algorithm did not identify any focus of adenocarcinoma missed by the pathologist in this study set. However, the algorithm did identify a number of very small “suspicious” foci. When the reviewers were asked to manually re-review previously reviewed images, but this time specifically knowing that the algorithm had called them “suspicious”, and with the algorithm highlighting the focus it called suspicious, in about a half of these images at least one of the two reviewers changed their diagnosis from benign to glandular atypia. Given, as stated above, that lesions such as these can prompt additional studies by the pathologist as they can be associated with adenocarcinoma on re-biopsy, the use of Paige Prostate to identify such lesions might prove a useful adjunct to pathologists’ review. In this study, the “suspicious discrepant” core biopsies came from 28 different patients, 23 of whom had cancer in at least one other core of the case; the prevalence of carcinoma in these patients was 82%. This prevalence rate is somewhat higher than the 73% prevalence rate of cancer among all of the patients in the study. These data suggest that Paige Prostate is finding even very small foci of atypical glands. Finally, our study was designed to assess the performance of Paige Prostate on a cohort that is representative of our practice. However, the possibility that our unbalanced cohort favors a diagnosis of “not suspicious” and thus impacts the performance metrics of the algorithm cannot be ruled out.

Paige Prostate has been shown to increase detection of prostate cancer in whole-slide images when reviewed by non-GU subspecialized pathologists [13]. In a highly subspecialized practice setting such as Yale Pathology, where many prostate biopsies are reviewed by at least one GU subspecialized pathologist, possibly more than once, the benefit of Paige Prostate to improve on sensitivity of diagnosis may be limited; however, further studies are needed to investigate whether Paige Prostate, or similar tools, can improve the efficiency of slide review and how its use might influence the utilization of ancillary studies (i.e., immunohistochemical stains, levels) and consensus review. Another important factor which may have a bearing on these results is that, since Yale is a tertiary referral center, the prevalence of malignancy was quite high in this study set: 26% of the biopsies and 73% of the patients. This is higher than the prevalence rate in most community hospital settings.

The study also highlights areas for possible improvement of the algorithm. For example, better identification of out-of-focus scans would decrease the false negative rate. On the other hand, ignoring images containing only a single focus less than 0.25 mm would decrease the false positive rate for the algorithm. Building in functionality for Gleason grading and automatically providing the pathologist with measurement data to automate aspects of the College of American Pathologists reporting protocols such as the total length of the biopsy, the maximum length of the cancer, and the percentage involvement are additional enhancements that would increase the utility of the tool in practice. Overall, Paige Prostate shows potential to be a versatile tool with varied use-case applications in anatomic pathology practice settings.

## Supplementary information

Supplementary Figure 1

## Data Availability

The datasets used and/or analyzed during the current study are available from the corresponding author on reasonable request.
