# Supplementary material for: An independent assessment of an artificial intelligence system for prostate cancer detection shows strong diagnostic accuracy
Source: Mod Pathol. 2021 Mar 29;34(8):1588–95. doi: 10.1038/s41379-021-00794-x (PMC8295034; doi:10.1038/s41379-021-00794-x)
Supplement: Supplementary file 1 — Supplementary Figure 1 [file 41379_2021_794_MOESM1_ESM.docx]

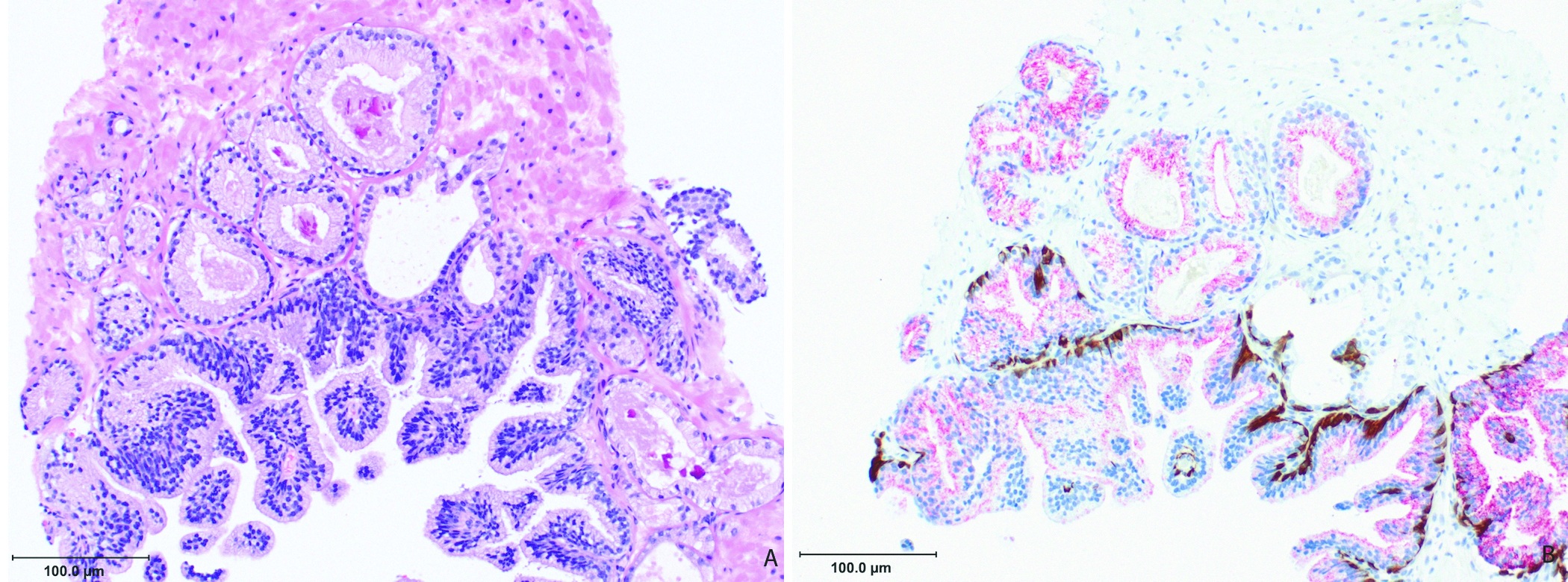


Supplementary figure 1. Core biopsy showing adenocarcinoma with foamy gland features (Suppl. Fig 1A) and confirmatory PIN-4 immunohistochemical stain (Suppl. Fig 1B) that was correctly flagged as suspicious by Paige Prostate. (Scale bars =100μm).
